# Adiponectin Protects against Glutamate-Induced Excitotoxicity via Activating SIRT1-Dependent PGC-1α Expression in HT22 Hippocampal Neurons

**DOI:** 10.1155/2016/2957354

**Published:** 2016-11-30

**Authors:** Liang Yue, Lei Zhao, Haixiao Liu, Xia Li, Bodong Wang, Hao Guo, Li Gao, Dayun Feng, Yan Qu

**Affiliations:** Department of Neurosurgery, Tangdu Hospital, Fourth Military Medical University, Xi'an, China

## Abstract

Glutamate- (Glu-) induced excitotoxicity plays a critical role in stroke. This study aimed to investigate the effects of APN on Glu-induced injury in HT22 neurons. HT22 neurons were treated with Glu in the absence or the presence of an APN peptide. Cell viability was assessed using the MTT assay, while cell apoptosis was evaluated using TUNEL staining. Levels of LDH, MDA, SOD, and GSH-Px were detected using the respective kits, and ROS levels were detected using dichlorofluorescein diacetate. Western blot was used to detect the expression levels of silent information regulator 1 (SIRT1), peroxisome proliferator-activated receptor gamma coactivator 1-alpha (PGC-1*α*), cleaved caspase-3, Bax, and Bcl-2. In addition to the western blot, immunofluorescence was used to investigate the expression levels of SIRT1 and PGC-1*α*. Our results suggest that APN peptide increased cell viability, SOD, and GSH-Px levels and decreased LDH release, ROS and MDA levels, and cell apoptosis. APN peptide upregulated the expression of SIRT1, PGC-1*α*, and Bcl-2 and downregulated the expression of cleaved caspase-3 and Bax. Furthermore, the protective effects of the APN peptide were abolished by SIRT1 siRNA. Our findings suggest that APN peptide protects HT22 neurons against Glu-induced injury by inhibiting neuronal apoptosis and activating SIRT1-dependent PGC-1*α* signaling.

## 1. Introduction

Stroke has been ranked as one of the most common diseases that can lead to high mortality and disability [1, 2]. There are many studies showing that the incidence of stroke is increasing worldwide [3–5]. There are multiple causes of neuronal injury in stroke. Among these, excessive release of glutamate (Glu) and the accompanying oxidative stress play significant roles. They may lead to neuronal damage through receptor-mediated excitotoxicity, which plays an important role in many central nervous system diseases and their pathologic process [6–8]. Oxidative stress induced by overexcitation has been considered to trigger a series of intracellular reactions including activation of death receptor signaling pathway, dysfunction of mitochondria, and loss of antiapoptotic effects and finally cause cell death [9–11]. Therefore, finding an effective way to prevent this could be a reasonable method to protect the brain from stroke injury.

Adiponectin (APN) is an adipokine that is almost completely secreted by adipocytes [12, 13]. It has been shown that APN can increase the sensitivity of peripheral tissues to insulin and has antidiabetic and antiatherogenic effects [13–15]. Further, much evidence suggests that APN plays an effective role in cardioprotection and neuroprotection in ischemia/reperfusion injury [16–19]. In terms of how APN attenuates excitotoxicity and brain damage in acute cerebral ischemia, the detailed mechanisms remain to be explored. Even though human-derived APN has been successfully synthesized, it is hard to investigate its neuroprotective effects through intravenous injection, as its relatively large size prevents it from passing through the blood-brain barrier. Due to this setback, APN knockout mice, adenovirus-mediated supplementation of APN, and intracerebroventricular injection of APN have been the primary methods of previous research [17, 20, 21]. In the present study, we used a variant of APN peptide that was synthesized according to the APN amino acid sequence and has been shown to simulate APN function. This peptide was patented by the State Intellectual Property Office of the People's Republic of China, and it has been shown that, after intraperitoneal injection or tail intravenous injection in mice, this APN peptide can pass through the blood-brain barrier and combine with the intracephalic APN receptor and act similarly to APN.

Silent information regulator 1 (SIRT1) is a nicotinamide-adenine dinucleotide-dependent histone deacetylase [22]. Many studies suggest that SIRT1 can take part in cell metabolism, proliferation, senescence, lesion, and repair, and SIRT1 plays a critical role in antiapoptosis and anti-inflammation through its direct or indirect effects [23–26]. A previous study of ours suggests that the activation of SIRT1 can attenuate inflammation, apoptosis, and oxidative stress in sepsis-induced brain injury [27]. Notably, one study on the progression of pancreatic cancer showed that APN can protect pancreatic beta cells against apoptosis via activation of 5′ AMP-activated protein kinase (AMPK), SIRT1, and peroxisome proliferator-activated receptor gamma coactivator 1-alpha (PGC-1*α*) signaling [28]. Furthermore, modulating the APN receptor 1 (AdipoR1) and inducing AMPK/SIRT1 activation can improve neuropathological deficits in Alzheimer's disease [29]. However, the exact role of SIRT1 in the cytoprotective effects of APN is still not completely understood, especially in cases of stroke. In this study, we aimed to elucidate the connection between APN and SIRT1/PGC-1*α* pathways and their roles in Glu-induced oxidative stress in brain damage.

## 2. Materials and Methods

### 2.1. Reagents

Adiponectin peptide was entrusted and synthesized by Sangon Biotech Co., Ltd (Shanghai, China). Glutamate, dichlorofluorescin diacetate (DCF-DA), and 4′,6-diamino-2-phenylindole (DAPI) were purchased from Sigma-Aldrich (St. Louis, MO, USA). 3-(4,5-Dimethylthiazol-2-yl)-2, 5-diphenyltetrazolium bromide (MTT) and dimethyl sulfoxide (DMSO) were purchased from Beyotime (Shanghai, China). The assay kits of LDH, MDA, SOD, and GSH-Px were purchased from Nanjing Jiancheng Bioengineering Institute (Nanjing, China). Terminal deoxynucleotidyl transferase dUTP nick-end labeling (TUNEL) kits were purchased from Roche (Mannheim, Germany). Antibodies against SIRT1, cleaved caspase-3, Bax, Bcl-2, and *β*-actin were purchased from Cell Signaling Technology (Beverly, MA, USA). Antibody against PGC-1*α* was purchased from Santa Cruz Biotechnology (Santa Cruz, CA, USA). The appropriate secondary antibodies were purchased from Beyotime (Shanghai, China). SIRT1 siRNA and control siRNA were purchased from Santa Cruz Biotechnology (Santa Cruz, CA, USA).

### 2.2. Cell Culture and Treatments

Mouse HT22 hippocampal cells were cultured in DMEM medium (GE Healthcare Life Sciences, Logan, UT, USA) containing 10% (v/v) fetal calf serum (FBS) and 0.35% glucose and supplemented with duplex antibiotics of 100 U/mL penicillin and 100 *μ*g/mL streptomycin, in a humidified atmosphere of 5% CO_2_ and 95% air at 37°C. The APN peptide was dissolved in dimethyl sulfoxide (DMSO) and diluted with culture medium immediately prior to use. DMSO (0.01%) was used as a sham control. The cells were pretreated with different concentrations of the APN peptide for 6 h before being exposed to Glu. The concentrations and durations of Glu exposure in the Glu group were selected based on a previous study [30]. After relevant treatments, cells were collected for further analysis.

### 2.3. Cell Viability Assay

The MTT assay was used to measure cell viability. Mouse HT22 hippocampal cells were cultured at a density of 5 × 10^4^/well in 96-well plates and treated with the previously mentioned method. The MTT assay was executed with 0.5 mg/mL MTT solution (20 *μ*L/well) for 4 h at 37°C and then DMSO (150 *μ*L/well) addition. Absorbance was measured at 490 nm using a SpectraMax M2 spectrometer (Molecular Devices, Sunnyvale, CA, USA). Optical density (OD) was used as our metric for cell viability. Cell viability was calculated using the following formula: cell viability (in %) = (mean optical density in test wells)/(mean optical density in control wells) × 100.

### 2.4. Lactate Dehydrogenase (LDH) Assay

A diagnostic kit was used to detect cellular lactate dehydrogenase (LDH) release into the culture medium and the process was according to the manufacturer's instructions (Jiancheng Bioengineering Institute, Nanjing, China). Briefly here, cells were treated with the way as mentioned above and 20 *μ*L of supernatant from each well was collected and incubated with the reduced form of nicotinamide-adenine dinucleotide and pyruvate for 15 min at 37°C, after which the reaction was stopped by adding 0.4 M NaOH. The activity of LDH was calculated from the absorbance at 440 nm. Background absorbance from culture medium that was not used for any cell cultures was subtracted from all absorbance measurements. All data are shown as fold changes versus the control.

### 2.5. Cellular Apoptosis Analysis

Cellular apoptosis was analyzed by performing a TUNEL assay using an in situ cell death detection kit (Roche, Mannheim, Germany) following the manufacturer's instructions. After 4% paraformaldehyde fixation and 0.1% Triton X-100 permeabilization, cells were incubated with 50 *μ*L TUNEL reaction mixture for 60 min at 37°C in the dark and then rinsed with PBS (pH 7.4) three times for 5 min each. Then after a 15 min DAPI counterstain in room temperature, cells were photographed with a fluorescence microscope. Apoptotic cell nuclei were stained by the TUNEL assay with green, and all nuclei were stained by DAPI with blue. The apoptotic index (in each image) (in %) = (positively stained apoptotic cells)/(total number of cells) × 100. Average number of three images from each group was presented as the final results.

### 2.6. Measurement of Intracellular Reactive Oxygen Species (ROS) Production

After various treatments, cells were incubated with dichlorofluorescein diacetate (DCF-DA, 10 *μ*M) for 30 min at 37°C in the dark and then washed with phosphate-buffered saline (PBS). A fluorescence microscope (FLX800, BioTek Instruments Inc., Winooski, VT, USA) and a Flex Station 3 fluorometric plate reader (Molecular Devices, Sunnyvale, CA, USA) were used to take photograph and record results with an excitation wavelength of 488 nm and an emission wavelength of 525 nm. Values are expressed as percentages of the fluorescence of the control group.

### 2.7. Determination of Lipid Peroxidation Products and Antioxidant Enzyme Activity

After a 30 min lysis on ice, cells were centrifuged at 12,000 ×g for 10 min at 4°C and the supernatants were collected for later experiments. Briefly, MDA was evaluated using the thiobarbituric-acid-reactive substance method. The cellular antioxidant enzyme (SOD and GSH-Px) activity was measured according to the manufacture's instruction of colorimetric assay kit (Nanjing Jiancheng Bioengineering Institute).

### 2.8. Immunofluorescence Assay

First, cells were fixed in 4% paraformaldehyde for 20 min. Then after a 10 min permeabilization within 0.1% Triton X-100 and blocking in 5% bovine serum albumin (BSA) for 30 min at room temperature, cells were incubated with anti-SIRT1 rabbit polyclonal antibodies (1 : 200) and anti-PCG1*α* rabbit polyclonal antibodies (1 : 100) overnight at 4°C. Then the cells were washed thrice in PBS for 5 minutes each and incubated with TRITC-conjugated goat anti-rabbit secondary antibodies (1 : 200) for 2 h at room temperature. After subsequently incubating with DAPI (0.02 mg/mL) for 10 min and rinsing with PBS, cells were wet-mounted using glycerol (50% v/v) and photographed using a fluorescence microscope (BX51, Olympus, Japan) with a charge-coupled device camera (DP70, Olympus, Japan).

### 2.9. Western Blotting

Western blots were performed as described previously [27]. Protein (20 *μ*g/lane) was separated using sodium dodecyl sulfate polyacrylamide gel electrophoresis (SDS-PAGE) and transferred to Immobilon nitrocellulose (NC) membranes (Merck Millipore, Billerica, MA, USA). After blocking with 5% nonfat milk in Tris-buffered saline and Tween 20 (TBST, pH 7.6), the membranes were incubated with primary antibodies against SIRT1, PGC-1*α*, cleaved caspase-3, Bax, Bcl-2, and *β*-actin (all 1 : 1000) overnight at 4°C. Then, the membranes were incubated with the appropriate horseradish peroxidase-conjugated secondary antibodies (1 : 5000) at room temperature for 1.5 h and then washed with TBST 3 times for 5 minutes each; the protein bands were detected using a Bio-Rad imaging system (Bio-Rad Laboratories, Hercules, CA, USA) and quantified using the Quantity One software package (Bio-Rad Laboratories). The value for the control group was defined as 100%.

### 2.10. Small RNA Interference

The sequences of small interfering RNA (siRNA) targeting mouse SIRT1 (5′-GGGAUCAAGAGGUUGUUAATTUUAACAACCUCUUGAUCCCTT-3′) were synthesized as previously reported [31]. HT22 cells (5 × 10^4^/well) were cultured in six-well plates and cultivated overnight for attachment. Then, the cells were transfected with scrambled siRNA or SIRT1-targeted siRNA (90 pM) using Lipofectamine 2000 (Thermo Fisher Scientific, Waltham, MA, USA) for 1 h in antibiotic-free medium. Then cells were rinsed in warm medium and incubated for 24 h in order to construct expression. After transfection and different treatments, cells were gathered and prepared for the relevant experiments.

### 2.11. Statistical Analysis

Data are presented as mean ± standard deviation (SD) and analyzed using SPSS 18.0 (IBM Corporation, Armonk, NY, USA) by one-way analysis of variance (ANOVA) followed by Scheffé's test for post hoc analysis. *P* < 0.05 was considered statistically significant.

## 3. Results

### 3.1. Effect with Different Concentration of Adiponectin Peptide on HT22 Cells

**Figure 1:**
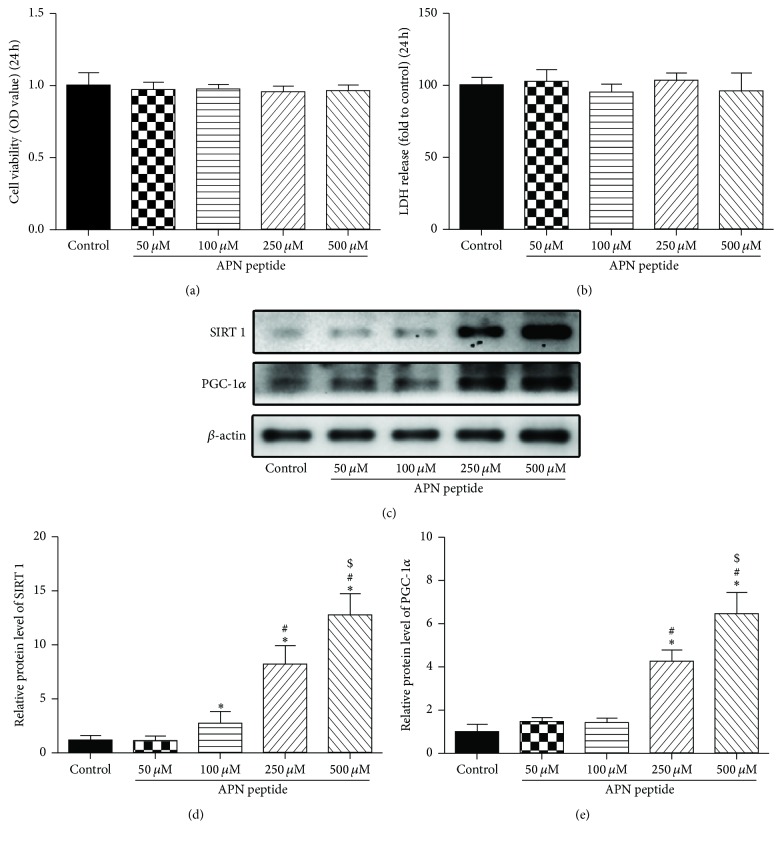
Effects of different concentrations of the APN peptide on HT22 cells. (a) HT22 cells were incubated with the APN peptide at different concentrations (50 *μ*M, 100 *μ*M, 250 *μ*M, or 500 *μ*M) for 24 h. Cell viability is shown in OD. (b) LDH release detection after treatments with indicated concentrations of APN peptide. The results are expressed as fold increase of the control levels. (c) SIRT1 and PGC-1*α* levels were analyzed by western blotting, and *β*-actin was used as the control protein. (d) SIRT1 expression level. (e) PGC-1*α* expression level. The data are presented as the mean ± SD. *n* = 6; ^*∗*^ *P* < 0.05 versus the control group; ^#^ *P* < 0.05 versus the 100 *μ*M APN peptide treatment group; ^$^ *P* < 0.05 versus the 250 *μ*M APN peptide treatment group; APN, adiponectin; OD, optical density; LDH, lactate dehydrogenase; SIRT1, silent information regulator 1; PGC-1*α*, peroxisome proliferator-activated receptor gamma coactivator 1-alpha.

First, we evaluated the effects of different concentrations of the APN peptide (50 *μ*M, 100 *μ*M, 250 *μ*M, and 500 *μ*M) on HT22 cells using the MTT assay. After being treated by APN peptide for 24 h the cell viability, expressed in OD, was analyzed. It showed that there were no significant differences for any of the APN groups compared with the control group (Figure 1(a)). We analyzed the LDH release of each group and found that the result was similar to the MTT assay (Figure 1(b)). We also detected SIRT1 and PGC-1*α* levels after pretreatment with the APN peptide at different concentrations and observed that levels of both proteins showed trends of gradual increase (*P* < 0.05; Figure 1(c)). We, thus, selected three concentrations (100 *μ*M, 250 *μ*M, and 500 *μ*M) of the APN peptide for further experiments.

### 3.2. Protective Effects of the APN Peptide against Glu-Induced Injury in HT22 Cells

**Figure 2:**
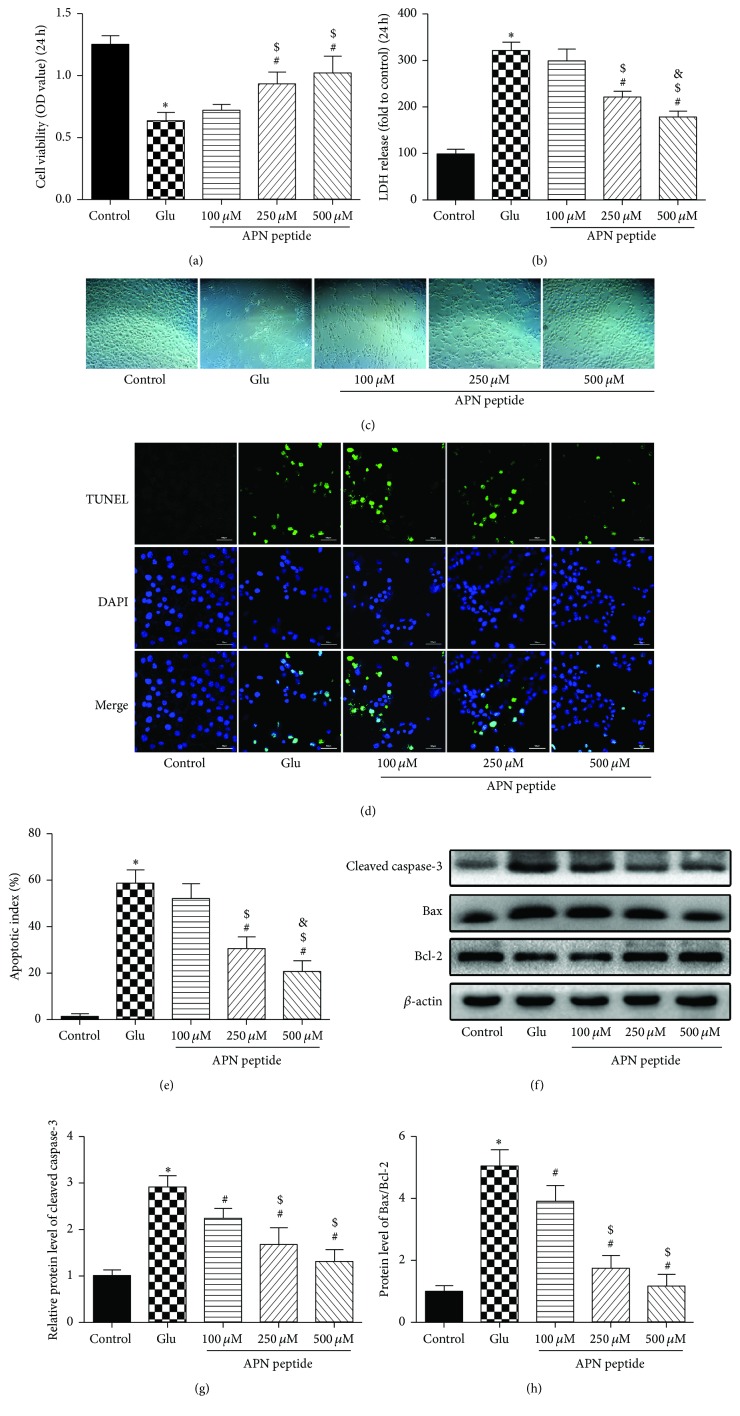
Protective effects of the APN peptide against Glu-induced injury in HT22 cells. (a) Cells were pretreated by the APN peptide with the mentioned concentrations above for 6 h and then exposed to 5 mM Glu for 24 h. Cell viability is shown in OD. (b) LDH release is expressed as fold increase of the control levels. (c) Cell images were taken using a light microscope. Cells shrank and showed decreased cell junctions in the Glu group but a gradual recovery following the increased concentrations of APN peptide in the APN peptide pretreatment groups. (d) HT22 cells were exposed (or not) to an APN peptide pretreatment at the indicated concentrations (100 *μ*M, 250 *μ*M, and 500 *μ*M) for 6 h followed by 5 mM Glu treatment for 24 h and then stained with TUNEL and DAPI. Representative images of apoptotic neurons (400x) were shown. The apoptotic cells were detected by TUNEL (green), and the nuclei were detected by DAPI (blue). (e) The apoptosis index showed a dose-dependent decrease in cell apoptosis. (f) Cleaved-caspase-3, Bax, and bcl-2 levels were analyzed by western blotting and *β*-actin was used as the control protein. (g) Cleaved caspase-3 expression level. (h) Bax/Bcl-2 ratio. The data are presented as the mean ± SD. *n* = 6; ^*∗*^ *P* < 0.05 versus the control group; ^#^ *P* < 0.05 versus the Glu group; ^$^ *P* < 0.05 versus the 100 *μ*M APN peptide pretreatment group; ^&^ *P* < 0.05 versus the 250 *μ*M APN peptide pretreatment group. APN, adiponectin; OD, optical density; LDH, lactate dehydrogenase.

A previous study showed that, after 24 h of treatment with 5 mM Glu, HT22 cell viability was reduced by more than 50% [30]; therefore, in this study, we used the same concentration in the Glu group. The MTT results showed that the OD in the Glu group was significantly reduced compared with the control group (*P* < 0.05; Figure 2(a)). However, along with morphological changes seen under the light microscope, after a 6 h pretreatment of 500 *μ*M APN peptide, cell viability increased significantly. The protective effects of 100 *μ*M and 250 *μ*M APN peptide were not as strong as 500 *μ*M but still slightly increased cell viability, and the 250 *μ*M group showed a significant difference compared to the Glu group (Figures 2(a) and 2(c); *P* < 0.05). In addition, the last two concentrations (250 *μ*M and 500 *μ*M) of the APN peptide effectively restrained increasing LDH release from Glu-induced damage in HT22 cells (Figure 2(b); *P* < 0.05). Next, we used a TUNEL assay to observe the effects of APN against Glu-induced apoptosis. As expected, high levels of the APN peptide could effectively attenuate the apoptosis induced by Glu in HT22 cells (*P* < 0.05; Figure 2(d)). After Glu treatment, the cellular apoptotic index significantly increased to 58.67 ± 5.68%. With the addition of a 6 h APN peptide pretreatment, however, the cell apoptotic index showed a dose-dependent decrease, and, with APN peptide concentration of 500 *μ*M, the apoptotic index significantly decreased to 20.33 ± 4.76% (*P* < 0.05, compared with the Glu group, Figure 2(e)). At the same time, we measured bcl-2, Bax, and cleaved caspase-3 levels in each group and found that APN peptide treatment downregulated the expression of cleaved caspase-3 and decreased the ratios of Bax and bcl-2 compared with the Glu group (*P* < 0.05, Figure 2(f)).

### 3.3. Effects of the APN Peptide on Glu-Induced Oxidative Stress

**Figure 3:**
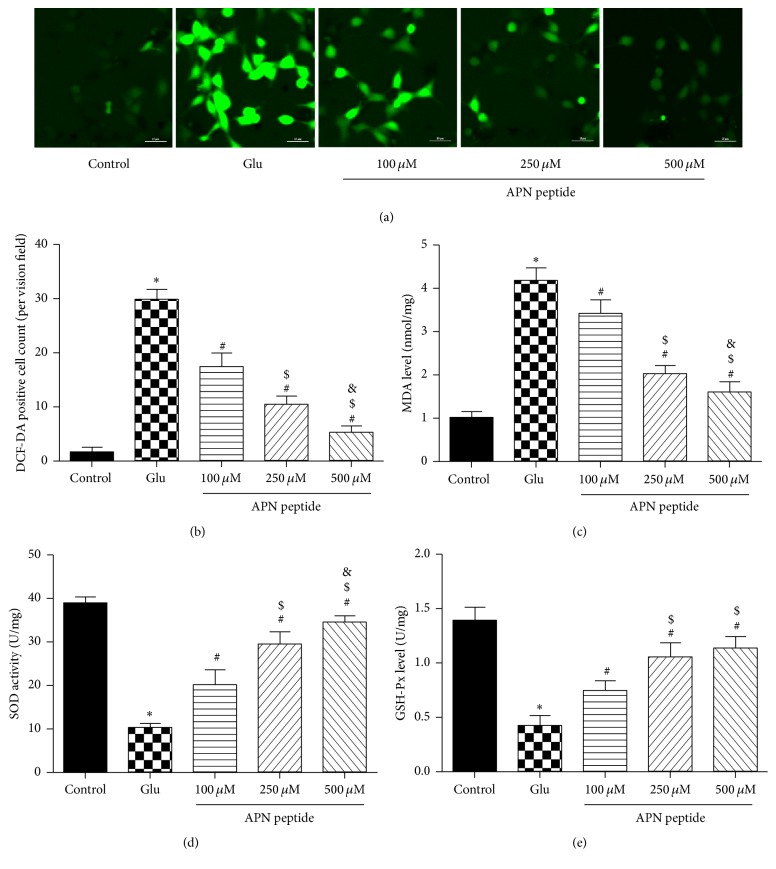
The APN peptide decreased Glu-induced oxidative stress. (a) HT22 cells were treated with the APN peptide at the indicated concentrations (100 *μ*M, 250 *μ*M, and 500 *μ*M) for 6 h and then exposed to 5 mM Glu for 24 h, followed by incubation with 10 *μ*M DCF-DA for 30 min. ROSs were stained with green fluorescence. (b) ROS levels. (c) MDA levels. (d) SOD activities. (e) GSH-Px levels. The results are expressed as the mean ± SD. *n* = 6; ^*∗*^ *P* < 0.05 versus the control group; ^#^ *P* < 0.05 versus the Glu group; ^$^ *P* < 0.05 versus the 100 *μ*M APN peptide pretreatment group; ^&^ *P* < 0.05 versus 250 *μ*M APN peptide pretreatment group. APN, adiponectin; OD, optical density; LDH, lactate dehydrogenase; ROS, reactive oxygen species.

We used the DCF-DA staining assay to assess the effects of the APN peptide on intracellular ROS levels. After Glu treatment, ROS generation markedly increased. However, with pretreatment of the APN peptide, ROS generation decreased significantly (*P* < 0.05; Figures 3(a) and 3(b)). This result suggests that APN decreases Glu-induced ROS production. Glu treatment dramatically decreased intracellular GSH-Px levels and SOD enzymatic activity but increased MDA levels compared to the control group (*P* < 0.05; Figures 3(c), 3(d), and 3(e)). However pretreatment with APN peptide for 6 h can reverse this trend on lipid peroxidation products and antioxidant enzyme activity (*P* < 0.01; Figures 3(c), 3(d), and 3(e)).

### 3.4. The Role of SIRT1 and PGC-1*α* in APN Peptide Protection against Glutamate-Induced Injury

**Figure 4:**
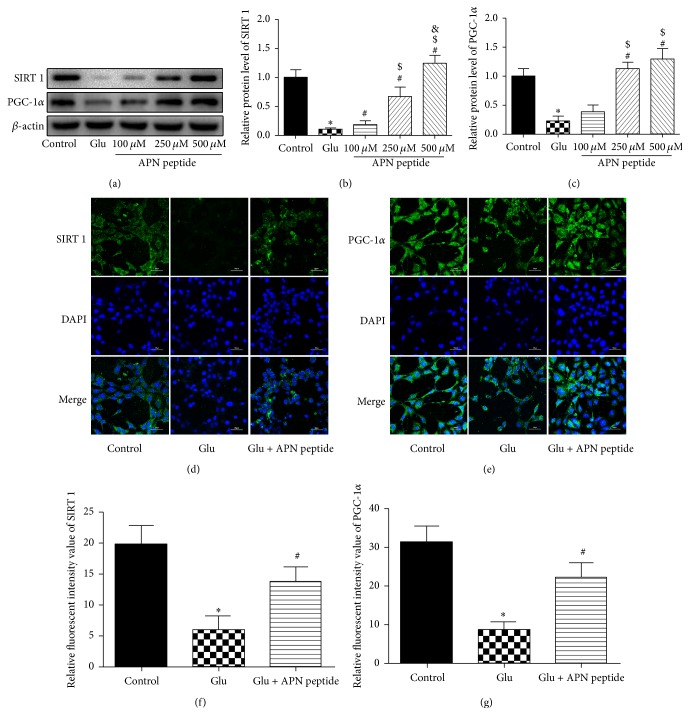
APN peptide pretreatment increased the expression of SIRT1 and PGC-1*α*. (a, b, and c) Western blot analysis of the effects of the APN peptide on SIRT1 and PGC-1*α* expression in HT22 cells exposed to Glu. (d and e) Immunofluorescence images of SIRT1 (green)/DAPI (blue) and PGC-1*α* (green)/DAPI (blue) stained neuronal cells after 6 h pretreatment of the APN peptide (500 *μ*M) and exposure to 5 mM Glu for 24 h. (f) SIRT1 expression level. (g) PGC-1*α* expression level. The results are expressed as the mean ± SD. *n* = 6; ^*∗*^ *P* < 0.05 versus the control group; ^#^ *P* < 0.05 versus the Glu group; ^$^ *P* < 0.05 versus the 100 *μ*M APN peptide pretreatment group; ^&^ *P* < 0.05 versus 250 *μ*M APN peptide pretreatment group. APN, adiponectin; SIRT1, silent information regulator 1; PGC-1*α*, peroxisome proliferator-activated receptor gamma coactivator 1-alpha.

We also analyzed expression levels of SIRT1 and PGC-1*α* with western blot and found that the expression of SIRT1 and PGC-1*α* was attenuated in the Glu group, but a recuperative increase in the APN peptide pretreatment group was observed (*P* < 0.05; Figures 4(a), 4(b), and 4(c)). Additionally, the expression of SIRT1 and PGC-1*α* was evaluated by immunofluorescence and the concentration of 500 *μ*M for APN peptide was selected. After the corresponding processing shown in Figures 4(d) and 4(e), the Glu group showed decreased expression of SIRT1 and PGC-1*α* compared with the control group. However, in the high-dosage APN peptide pretreatment group, the expression of both proteins increased significantly.

### 3.5. Effects of SIRT1 siRNA on APN Peptide Protection against Glu-Induced Injury

**Figure 5:**
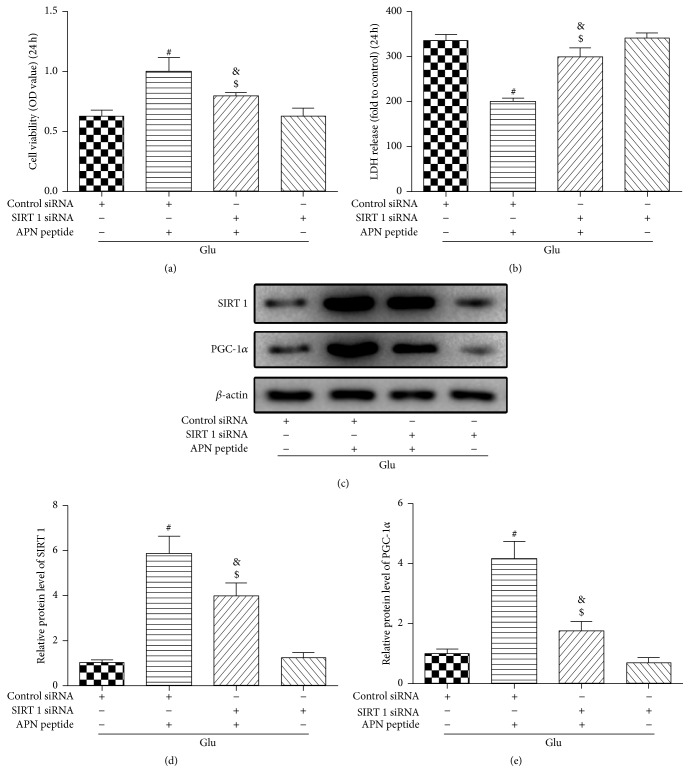
SIRT1 siRNA inhibited the expression of SIRT1 and PGC-1*α* and restrained the protective effects of the APN peptide against Glu-induced damage in HT22 cells. HT22 cells were transfected with siRNA targeting SIRT1 or negative control siRNA for 24 h and then exposed to 500 *μ*M APN peptide for 24 h, followed by exposure to 5 mM Glu for 24 h. (a) Cell viability is shown in OD. Silencing SIRT1 reversed the protective effects of the APN peptide against Glu-induced HT22 cell death. (b) LDH release was tested by a LDH release assay and the results are expressed as multiples of the control levels. Silencing SIRT1 also weakened the effects of the APN peptide and decreased Glu-induced LDH release. (c) Western blot analysis of SIRT1 and PGC-1*α* expressions after siRNA transfection; the cellular levels of SIRT1 and PGC-1*α* both decreased significantly. (d) SIRT1 expression level. (e) PGC-1*α* expression level. The results are expressed as mean ± SD. *n* = 6; ^#^ *P* < 0.05 versus the Glu + control siRNA group; ^$^ *P* < 0.05 versus the 500 *μ*M APN peptide + control siRNA group; ^&^ *P* < 0.05 versus the Glu + SIRT1 siRNA group. APN, adiponectin; OD, optical density; LDH, lactate dehydrogenase; SIRT1, silent information regulator 1; PGC-1*α*, peroxisome proliferator-activated receptor gamma coactivator 1-alpha.

We chose to silence SIRT1 using siRNA to further investigate the role of SIRT1 and the correlation between SIRT1 and PGC-1*α*. In this step, we selected one optimal protective concentration of the APN peptide (500 *μ*M) and transfected cells with SIRT1 siRNA for 24 h after APN peptide treatment. Cell viability and LDH release were evaluated, and the cells were harvested to detect protein expression by western blotting. As shown in Figures 5(a) and 5(b), even though SIRT1 could enhance cell viability against Glu-induced injury and reduce the cellular LDH release, the addition of SIRT1 siRNA restricted this function. Further, western blotting showed that after SIRT1 siRNA transfection in the Glu group, the cellular levels of SIRT1 and PGC-1*α* both showed a decrease. Moreover, compared to the control siRNA + APN peptide + Glu group, SIRT1 siRNA significantly downregulated the expression of SIRT1 and PGC-1*α*, which increased due to the APN peptide pretreatment (*P* < 0.05; Figures 5(c), 5(d), and 5(e)).

## 4. Discussion

Oxidative stress is one of the earliest events induced by ischemic injury in stroke and its cascade of cellular and molecular events is common in neural damage and cell death [32]. Many researchers have aimed to find some useful compounds and phytochemicals against neural cell damage caused by stroke, but the problems of bioavailability and side effects have restricted their success [33, 34]. Thus, there has been a growing interest in finding a safer, endogenous substance that is more easily absorbed by the human body.

Human APN, one of the members of the complement factor C1q family of proteins, is an adipocyte-derived hormone and its molecular weight is approximately 28 kDa. It circulates in high concentrations in healthy adults at around three times higher than leptin [35, 36]. There are several studies showing that APN has notable negative correlations with insulin resistance, and a decreased APN level is connected to reduced vascular function and leads to an increased risk of coronary artery disease [37, 38]. Some researchers have focused on its neuroprotective functions and have tried to reveal its detailed mechanisms. A recent study showed that APN can exert neurotrophic effects and neurogenesis in the dentate gyrus in male mice and suggests that APN plays an important role in hippocampal structural plasticity [39]. This indicates that exogenous APN supplements may be useful to improve hippocampal or neurological defects caused by ischemic stroke. However, due to the restricted permeability of the blood-brain barrier to macromolecules, the means of administration have become the problem. Based on the APN amino acid sequence and using protein analysis techniques and peptide synthesis technology, we developed a peptide that could reliably simulate and maintain the function of APN. The amino acid sequence of this synthetic peptide is NH2-LQVYGDGDHNGLYADNVN-COOH. After intraperitoneal injection in mice, this peptide could pass through the blood-brain barrier effectively and combine with the intracephalic APN receptors to play a similar role to endogenous APN. This peptide has been patented by the State Intellectual Property Office of the People's Republic of China with the patent number ZL201010185120.5. We chose this peptide to explore its actions in neurons in detail. In this study, the APN peptide played a protective role in murine hippocampal neuronal HT22 cells against Glu-induced oxidative stress damage in a concentration-dependent manner. It could restore cell viability and attenuate LDH release caused by Glu. Additionally, this peptide also protected against cell apoptosis and ROS generation caused by Glu-induced oxidative stress and improved the levels of GSH-Px and SOD while providing MDA levels relief.

SIRT1 has been reported to play an important role in many neurological diseases. From a cytological perspective, SIRT1 is correlated with mitochondrial function, endoplasmic reticulum stress, energy metabolism, the process of autophagy, apoptosis, and oxidative stress [22, 31, 40, 41]. Ota et al. reported that upregulation of SIRT1 can exert protective effects against cellular senescence and dysfunction in human endothelial cells [42]. In addition, Tsai et al. reported that oxidative stress-induced inhibition of SIRT1 function can promote 6-hydroxydopamine-induced cell death [43]. Notably, SIRT1 is also involved in cerebral ischemia. In in vivo experiments, mice overexpressing SIRT1 showed less hippocampal damage following bilateral common carotid artery occlusion compared to wild type mice [44]. Additionally, several endogenous or exogenous compounds shown to induce ischemic tolerance have also been linked to activation or upregulation of SIRT1 [45, 46]. Likewise, as an endogenous compound, APN also has the ability to activate SIRT1 and its downstream molecules [28], but in ischemic stroke it is unreported whether exogenous APN treatment has protective effects against oxidative stress damage via activation of SIRT1 and its downstream molecules. Moreover, as we have developed a new compound based on APN, the neurofunctional mechanisms of APN need to be clarified in detail before undertaking further trials. In the present study, single APN peptide treatments significantly increased SIRT1 expression, and the amount of protein expression gradually increased with treatment concentration. After cells suffered Glu-induced damage, SIRT1 expression was restrained, but pretreatment with the APN peptide could reverse this restriction and restore SIRT1 levels. As a regulator of mitochondrial biogenesis and function, PGC-1*α* plays a remarkable role in resistance to oxidative stress and mitochondrial integrity [47, 48]. There are several studies demonstrating that SIRT1 can enhance PGC-1*α* activation and make it act as a substrate of deacetylation [28, 49]. In the present study, we also detected PGC-1*α* levels, and its expression was dependent on the concentration of the APN peptide treatments. Importantly, after APN peptide pretreatment and Glu-induced damage, PGC-1*α* expression showed similar results to SIRT1. Further experiments with SIRT1 siRNA transfection showed that inhibition of SIRT1 can suppress the neuroprotective effects of the APN peptide. Further, SIRT1 siRNA transfection attenuated not only SIRT1 expression but also that of PGC-1*α*. These results suggest that SIRT1 influences the expression of PGC-1*α* and that the SIRT1/PGC-1*α* pathway may have a direct link with APN in fighting Glu-induced oxidative stress injury.

## 5. Conclusions

In summary, the present study demonstrates that APN has neuroprotective effects against Glu-induced excitotoxicity and oxidative stress injury in HT22 cells and acts via the SIRT1/PGC-1*α* signaling pathway. Further, this finding indicates that SIRT1 plays an important role in alleviating Glu-induced oxidative stress and neuron injury in cerebral ischemic stroke. These findings suggest that APN treatment may be a potential drug therapy aiming to treat cerebral ischemic stroke. We are excited to find that it may provide a new and effective way to deal with neurological deficiency and poor prognosis caused by ischemic stroke injury and provide new insight into the potential value of endogenous substances and their ramifications for neuroprotection. In further research, we plan to develop further in vivo studies and try to reveal the underlying molecular components and their involvement in these effects of APN.
